# Prostate volumetric‐modulated arc therapy: dosimetry and radiobiological model variation between the single‐arc and double‐arc technique

**DOI:** 10.1120/jacmp.v14i3.4053

**Published:** 2013-05-06

**Authors:** James C.L. Chow, Runqing Jiang

**Affiliations:** ^1^ Radiation Medicine Program Princess Margaret Cancer Center, University Health Network Toronto ON Canada; ^2^ Department of Radiation Oncology University of Toronto Toronto ON Canada; ^3^ Medical Physics Department Grand River Regional Cancer Center Kitchener ON Canada; ^4^ Department of Physics University of Waterloo Waterloo ON Canada

**Keywords:** prostate radiotherapy, VMAT, TCP, NTCP, treatment planning, dose‐volume histogram

## Abstract

This study investigates the dosimetry and radiobiological model variation when a second photon arc was added to prostate volumetric‐modulated arc therapy (VMAT) using the single‐arc technique. Dosimetry and radiobiological model comparison between the single‐arc and double‐arc prostate VMAT plans were performed on five patients with prostate volumes ranging from $29-68.1\;{cm}^{3}$. The prescription dose was 78 Gy/39 fractions and the photon beam energy was 6 MV. Dose‐volume histogram, mean and maximum dose of targets (planning and clinical target volume) and normal tissues (rectum, bladder and femoral heads), dose‐volume criteria in the treatment plan ($D_{99\%}$ of PTV; $D_{30\%},D_{50\%},V_{17Gy}$ and $V_{35Gy}$ of rectum and bladder; $D_{5\%}$ of femoral heads), and dose profiles along the vertical and horizontal axis crossing the isocenter were determined using the single‐arc and double‐arc VMAT technique. For comparison, the monitor unit based on the RapidArc delivery method, prostate tumor control probability (TCP), and rectal normal tissue complication probability (NTCP) based on the Lyman‐Burman‐Kutcher algorithm were calculated. It was found that though the double‐arc technique required almost double the treatment time than the single‐arc, the double‐arc plan provided a better rectal and bladder dose‐volume criteria by shifting the delivered dose in the patient from the anterior–posterior direction to the lateral. As the femoral head was less radiosensitive than the rectum and bladder, the double‐arc technique resulted in a prostate VMAT plan with better prostate coverage and rectal dose‐volume criteria compared to the single‐arc. The prostate TCP of the double‐arc plan was found slightly increased (0.16%) compared to the single‐arc. Therefore, when the rectal dose‐volume criteria are very difficult to achieve in a single‐arc prostate VMAT plan, it is worthwhile to consider the double‐arc technique.

PACS number: 87.55.D‐, 87.55.dk, 87.55.K‐, 87.55.Qr

## I. INTRODUCTION

In radical prostate radiotherapy, volumetric‐modulated arc therapy (VMAT) becomes a popular delivery option, taking advantage of shorter delivery time and smaller monitor unit (MU) compared to step‐and‐shoot intensity‐modulated radiotherapy (IMRT).^1^, ^2^, ^3^, ^4^, ^5^, ^6^ Patient dosimetry studies between prostate VMAT and IMRT showed that prostate VMAT can produce equivalent or even better target coverage and normal tissue (rectum, bladder and femoral heads) sparing.^7^, ^8^, ^9^, ^10^, ^11^ However, unlike step‐and‐shoot IMRT, prostate VMAT interplays more dose delivery parameters such as dynamic multileaf movement, dose rate, and gantry speed within a single or multiple photon arcs in the treatment.^12^, ^13^, ^14^, ^15^ This complex delivery technique, therefore, requires more dedicated machine and patient quality assurance procedure, MU calculation algorithm, and dosimetric evaluation (such as when patient size reduction due to weight loss) in the treatment.^16^, ^17^, ^18^, ^19^

Although single‐arc prostate VMAT has target coverage and dose homogeneity comparable to step‐and‐shoot IMRT, treatment planning dose‐volume criteria were sometimes difficult to achieve because of the complex geometry between the prostate and mobile rectum with irregular shape.^7^, ^11^, ^20^ To further reduce the rectal dose per planning dose‐volume criteria (e.g. $D_{30\%},D_{50\%},V_{17Gy}$ and $V_{35Gy}$), the double‐arc technique has to be employed to improve the target coverage and rectal sparing. The dosimetry of prostate VMAT using the single‐arc and double‐arc technique was studied by some groups. From a retrospective planning study, Guckenberger et al.^(7)^ compared the dose‐volume criteria among prostate step‐and‐shoot IMRT, single‐arc, and multiple‐arc VMAT. They concluded that the multiple‐arc prostate VMAT had a better dosimetric result than the single‐arc at a cost of increased delivery time, MU, and spread of low doses. Wolff et al.^(11)^ further compared the homogeneity and conformity index between the single‐arc and multiple‐arc (one 360° rotation plus 200° second rotation) prostate VMAT. They found that both indexes were higher for the multiple‐arc technique with a relatively longer delivery time (3.7 min) compared to the single‐arc (1.8 min). In the dosimetry comparison performed by Sze et al.,^(20)^ the authors reported that though the single‐arc technique was more efficient regarding the delivery time and MU, it resulted in a higher rectal dose compared to the double‐arc. They concluded that for a busy treatment unit, the single‐arc technique could be an acceptable option provided that all planning dose‐volume criteria were fulfilled.

In this study, apart from the dosimetry (dose‐volume criteria, mean and maximum dose) and MU comparison between the single‐arc and double‐arc technique, the reason of applying a second arc in the double‐arc technique was investigated, based on changes of dose distribution in different directions (left, right, anterior, and posterior). Moreover, prostate tumor control probability (TCP) and rectal normal tissue complication probability (NTCP) were calculated using the Lyman‐Burman‐Kutcher radiobiological model.^21^, ^22^, ^23^ The aim of this study is to investigate the dosimetry and radiobiological parameter variation between the single‐arc and double‐arc prostate VMAT. Results in this study should help medical physicists to understand the rationale of using more than one arc in the double‐arc prostate VMAT plan.

## II. MATERIALS AND METHODS

### A. Patient data

Computed tomography (CT) image dataset of five patients with localized prostate cancer were selected at the Grand River Hospital in this retrospective planning study. All CT‐simulations were carried out with patients in supine position and full bladder. The prostate volumes were in the range of 29 to $68.1\;{cm}^{3}$. The planning target volume (PTV), clinical target volume (CTV), rectum, bladder, and femoral heads of all patients were contoured by the same person. The gross target volume was equal to the CTV, and PTV was created by expansion of the CTV with 1 cm around, except 0.7 cm posteriorly. Details about the target and critical organ (rectum, bladder, and femoral heads) volumes can be found in Table 1.

**Table 1:** Prostate volumes, PTVs, critical organ volumes (rectum, bladder and femoral head), and monitor units of the single‐arc and double‐arc prostate VMAT plans for the five patients.

*Patient Number*	*Prostate Volume (cm* ^*3*^ *)*	*PTV (* ${cm}^{3}$ *)*	*Rectal Volume (* ${cm}^{3}$ *)*	*Bladder Volume (* ${cm}^{3}$ *)*	*Left Femoral Head Volume (* ${cm}^{3}$ *)*	*Right Femoral Head Volume (* ${cm}^{3}$ *)*	*Monitor Units*	
							*Single‐arc*	*Double‐arc*
1	29.0	107.0	42.3	182.5	146.6	153.4	550	598
2	39.7	134.1	110.1	379.5	190.4	189.0	519	676
3	54.8	170.4	80.1	552.6	201.4	246.0	471	587
4	57.3	176.7	50.2	243.9	199.7	193.7	600	610
5	68.1	196.1	76.8	184.5	180.0	198.2	540	730

### B. Treatment planning

Single‐arc and double‐arc prostate VMAT plans were created by the Eclipse treatment planning system (version 8.5, Varian Medical Systems, Palo Alto, CA) using the Progressive Resolution Optimizer (PRO) in the Rapid Arc optimization (Varian Medical Systems). The treatment planning system was commissioned for a Varian 21 EX linear accelerator (Varian Medical Systems) with a 120‐leaf Millennium multileaf collimator (MLC) and 6 MV photon beam. The dose constraints to critical organs, plan objectives, and optimization parameters of prostate VMAT plan can be found in our previous work.^(24)^ Dose calculations were performed using the Anisotropic Analytical Algorithm (ver. 8.9).^(25)^ Prostate VMAT plans were first created using the double‐arc technique for all patients. Then, the number of photon arc was reduced to one to generate the single‐arc plans for comparison. The calculated MU for the single‐arc and double‐arc plans can be found in Table 1. The average delivery times of the single‐arc and double‐arc prostate VMAT were 2.0 and 3.9 minutes, respectively, though the average MU of the double‐arc plan was only increased by about 20% (Table 1) compared to the single‐arc. Average dose‐volume histograms (DVHs), and mean and maximum doses of targets (PTV and CTV) and critical organs (rectum, bladder, and femoral heads) were determined. Moreover, mean dose‐volume criteria including the $D_{99\%}$ of PTV, $D_{30\%},D_{50\%},V_{17Gy}$, and $V_{35Gy}$ of rectum and bladder, and $D_{5\%}$ of femoral heads were calculated for both techniques.

### C. TCP and NTCP calculation

The prostate TCP was calculated as follows:

(1) $$TCP=\frac{\text{exp}(p+qD)}{1+\text{exp}(p+qD)}$$

*D* is dose, *p* and *q* are related to $D_{50}$ and $\gamma_{50}$ (normalized slope at the point of 50% probability control), according to Okunieff et al.^(26)^ who summarized clinical data for a variety of tumors that can be related to the slope and dose to control 50% of tumors. Using Eq. (1), control probability for the tumorlet with volume and doses, TCP ($v_{i},D_{i}$) can be inferred from the TCP for the whole volume by:

(2) $$TCP(v_{i},D_{i})=TCP{\left(D_{i}\right)}^{v_{i}}$$

where ($v_{i},D_{i}$) refers to the differential DVH converted from the cumulated DVH.

Rectal NTCP was calculated using the Lyman‐Burman‐Kutcher algorithm with the following equations:^21^, ^22^, ^23^

(3) $$NTCP=\frac{1}{2\pi }\int_{‐\infty }^{t}e^{‐\frac{x^{2}}{2}}dx$$

and

(4) $$t=\frac{D‐TD_{50}(v)}{mTD_{50}(v)}$$

where $v=V/V_{ref}$ and ${TD}_{50}(v)={TD}_{50}(1)v^{-n}$, as suggested by Burman et al.^(22)^ ${TD}_{50}=80\;Gy,n=0.12$, and m=0.15 were used to calculate the rectal NTCP in this study. Both TCP and NTCP were determined using an in‐house TCP/NTCP software running on a MATLAB platform (The MathWorks, Natick, MA).^(27)^

## III. RESULTS

**Figure 1:**
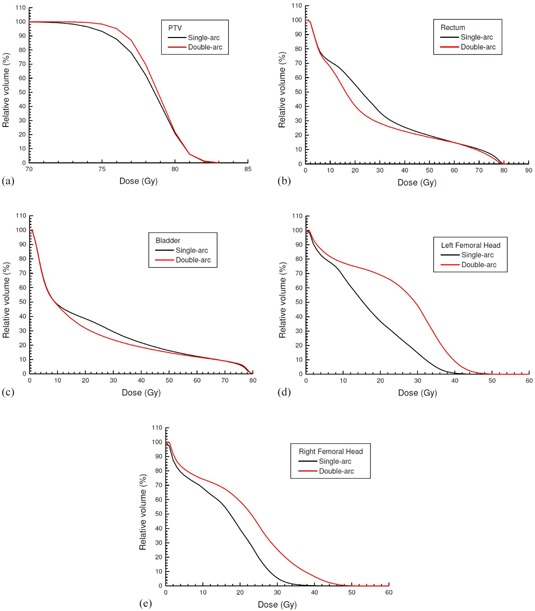
Average dose‐volume histograms of the (a) PTV, (b) rectum, (c) bladder, (d) left femoral head, and (e) right femoral head for the single‐arc and double‐arc prostate VMAT plans.

**Figure 2:**
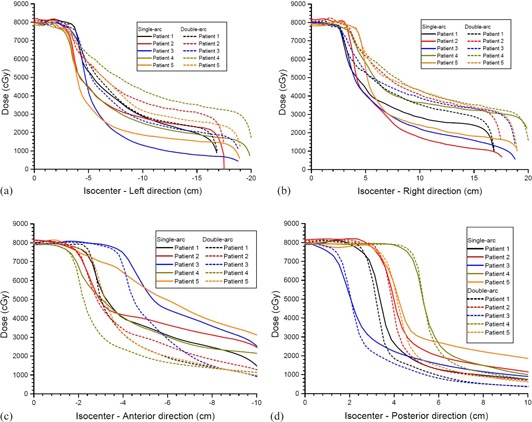
Dose profiles of the (a) left, (b) right, (c) anterior, and (d) posterior directions for the single‐arc and double‐arc prostate VMAT plans of five patients. The origin represents the isocenter. The negative values in the x‐axes represent the left and anterior direction.

**Figure 3:**
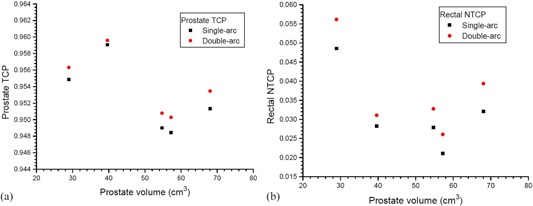
Prostate TCP (a) and rectal NTCP (b) varying with the prostate volume of the five patients based on the single‐arc and double‐arc prostate VMAT plans.

Average cumulated DVHs of the PTV, rectum, bladder, and left and right femoral head are shown in Figs. 1(a) to 1(e), planned using the single‐arc and double‐arc technique. The $D_{99\%}$ of PTV, $D_{30\%},D_{50\%},V_{17Gy}$, and $V_{35Gy}$ of rectum and bladder, and $D_{5\%}$ of left and right femoral head of the patients can be found in Table 2, which also shows the average mean and the maximum doses of targets (PTV and CTV) and critical organs using the two techniques. Dose profiles from the isocenter to the left, right, anterior, and posterior direction are plotted in Figs. 2(a) to 2(d) for all patients with the single‐arc and double‐arc technique. The prostate TCP and rectal NTCP are plotted against the prostate volume in Figs. 3(a) and 3(b), respectively.

**Table 2:** Mean dose‐volume criteria, and average mean and maximum doses of the PTV, CTV, and critical organs for the single‐arc and double‐arc prostate VMAT plans. The standard deviations are shown inside the brackets. $V_{17Gy}$ and $V_{35Gy}$ are percentage volumes receiving at least 17 Gy and 35 Gy, respectively. $D_{5\%},D_{30\%},D_{50\%}$, and $D_{99\%}$ are doses given to 5%, 30%, 50%, and 99% of volumes, respectively.

	*Mean Dose‐volume Criteria*	*PTV*	*CTV*	*Rectum*	*Bladder*	*Left Femoral Head*	*Right Femoral Head*
Single‐arc	$D_{99\%}$ (Gy)	72.5 (0.8)	–	–	–	–	–
	$D_{30\%}$ (Gy)	–	–	34.7 (6.1)	28.3 (8.4)	–	–
	$D_{50\%}$ (Gy)	–	–	22.3 (4.6)	10.6 (6.4)	–	–
	$D_{5\%}$ (Gy)	–	–	–	–	31.0 (6.6)	28.4 (4.9)
	$V_{35Gy}$ (%)	–	–	29.6 (5.9)	25.1 (5.3)	–	–
	$V_{17Gy}$ (%)	–	–	61.1 (8.5)	40.7 (9.8)	–	–
	Mean dose (Gy)	78.3 (0.7)	80.1 (0.7)	28.0 (3.9)	22.2 (4.3)	16.6 (4.0)	15.8 (1.8)
	Maximum dose (Gy)	82.7 (0.8)	82.6 (0.7)	80.1 (0.8)	80.7 (1.4)	40.4 (5.7)	38.9 (3.8)
Double‐arc	$D_{99\%}$ (Gy)	74.6 (0.4)	–	–	–	–	–
	$D_{30\%}$ (Gy)	–	–	28.5 (6.6)	22.2 (7.0)	–	–
	$D_{50\%}$ (Gy)	–	–	15.9 (3.1)	9.2 (3.7)	–	–
	$D_{5\%}$ (Gy)	–	–	–	–	41.6 (2.1)	39.4 (3.9)
	$V_{35Gy}$ (%)	–	–	25.1 (4.9)	20.8 (4.9)	–	–
	$V_{17Gy}$ (%)	–	–	47.7 (9.0)	35.4 (7.8)	–	–
	Mean dose (Gy)	78.7 (0.8)	80.3 (0.6)	25.1 (3.6)	20.4 (3.9)	24.9 (2.5)	21.1 (3.8)
	Maximum dose (Gy)	82.8 (0.7)	82.9 (0.8)	80.1 (0.8)	80.7 (1.1)	49.4 (2.4)	47.1 (5.8)

## IV. DISCUSSION

### A. Dose‐volume histogram

Figure 1(a) shows the average DVH of PTV for all patients planned using the single‐arc and double‐arc technique. The dose range in Fig. 1(a) is started from 70 Gy instead of zero to focus on the drop‐off region of the curve. It is seen in the figure that DVH curves of the double‐arc plans had a shaper drop‐off than those of the single‐arc for all patients. This result agrees with that found by other groups which have proved that the double‐arc technique can improve the dose conformity in the target volume.^11^, ^20^ Figures 1(b) and 1(c) show average DVHs of the rectum and bladder, respectively. It can be seen that percentage volumes receiving given doses (e.g., $V_{17Gy}$ and $V_{35Gy}$) were always lower in the double‐arc plan than the single‐arc. This shows that the double‐arc technique resulted in a better rectal and bladder dose‐volume criteria than the single‐arc. However, for average DVHs of the left and right femoral head in Figs. 1(d) and 1(e), it is found that the femoral head sparing became worse when the double‐arc technique was used compared to the single‐arc. Based on the results in Figs. 1(a) to 1(e), the double‐arc technique is found to improve the dose conformity and coverage of the prostate, and the rectal and bladder dose‐volume criteria. However, the cost is to worsen the left and right femoral head sparing.

### B. Dose‐volume criteria, maximum and mean dose

Mean dose‐volume criteria, maximum and mean dose are parameters important in the treatment plan evaluation. Table 2 shows the mean dose‐volume criteria of PTV, rectum, bladder, and femoral head calculated by the treatment planning system. In this study, the dose‐volume evaluation criteria for the prostate VMAT plan are: $D_{99\%}$ of PTV $\geq 74.1\;Gy,D_{30\%}$ of rectum and bladder $\leq 70\;Gy,D_{50\%}$ of rectum and bladder $\leq 53\;Gy,D_{5\%}$ of femoral head ≤53 Gy . For the PTV, it is seen in Table 2 that the mean $D_{99\%}$ of all patients (72.5 Gy) is less than 74.1 Gy based on the single‐arc technique. The double‐arc technique having higher mean $D_{99\%}$ of 74.6 Gy, on the other hand, satisfied the evaluation criteria. This shows that when the double‐arc technique was replaced by the single‐arc, $D_{99\%}$ of PTV would get worsen. For the mean $D_{30\%}$ and $D_{50\%}$ of the rectum and bladder, both the single‐arc and double‐arc technique satisfied the corresponding dose‐volume criteria. However, the double‐arc technique had lower $D_{30\%}$ and $D_{50\%}$ of rectum (on average 18% and 29%) than the single‐arc. The mean $D_{30\%}$ and $D_{50\%}$ of bladder were also found to be lower (on average 22% and 13%) when using the double‐arc technique compared to the single‐arc. For the left and right femoral head, the double‐arc technique had the mean $D_{5\%}$ (on average 34% and 39%) more than the single‐arc, but both techniques did not have the $D_{5\%}$ higher than the dose‐volume evaluation criteria of 53 Gy. For percentage rectal and bladder volume receiving at least a given dose, lower $V_{17Gy}$ and $V_{35Gy}$ of the rectum and bladder can be found when using the double‐arc technique compared to the single‐arc. In Table 2, it is seen that the double‐arc technique can effectively decrease the dose‐volume evaluation criteria for the rectum and bladder. However, the effect is increased doses in the left and right femoral head.

For the average mean and maximum doses of targets and critical organs (Table 2), when using the double‐arc technique, mean doses of the rectum and bladder decreased while those of the left and right femoral head increased. As can be seen in Table 2, the double‐arc technique increased the mean doses of the PTV and CTV insignificantly. For the maximum doses of targets and critical organs, no obvious trend of dose variation can be found when using the double‐arc technique. This shows that the maximum doses of targets and normal tissues are not sensitive to the number of photon arc in prostate VMAT.

### C. Dose profiles

To investigate how the double‐arc technique affects the dose distribution resulting in variations of dose‐volume criteria, average mean and maximum dose compared to the single‐arc dose profiles along the vertical and horizontal axis crossing at the isocenter were plotted, as shown in Fig. 2. It is seen in Figs. 2(a) and 2(b) that doses in the left and right direction were lower when the single‐arc technique was used instead of the double‐arc. In contrast, doses in the anterior (Fig. 2(c)) and posterior (Fig. 2(d)) direction for the double‐arc technique were lower than those of the single‐arc. From dose distributions of all patients along the vertical and horizontal axis, it can be seen that the addition of a second photon arc shifted the delivered dose from the anterior–posterior direction to the lateral direction. This resulted in lower dose‐volume criteria (e.g., $D_{30\%},D_{50\%},V_{17Gy}$, and $V_{35Gy}$) of the rectum and bladder in the anterior–posterior direction, but higher dose‐volume criteria ($D_{5\%}$) of the left and right femoral head in the left–right direction (Table 2). Since the increase of dose at the femoral head is within the normal tissue tolerance, the application of the double‐arc technique is simply to lower the rectal and bladder dose‐volume criteria at the cost of increasing the femoral head dose‐volume criteria within tolerance limit, so as to achieve a desired PTV coverage.

### D. Prostate TCP and rectal NTCP

The prostate TCP for the whole treatment (78 Gy/39 fractions) against the prostate volume is plotted in Fig. 3(a). It is seen in the figure that the prostate TCP for the double‐arc technique is slightly (0.16%) higher than that of the single‐arc. For NTCP of critical organs, since the bladder and femoral head NTCP are generally about $1\times 10^{2}$ and $1\times 10^{5}$ times smaller than the rectal NTCP, only the rectal NTCP is considered in this study.^28^, ^29^ It is found in Fig. 3(b) that the rectal NTCP for the double‐arc technique was higher than that of the single‐arc by about 17.5% on average. The reason is that in prostate VMAT, there is a high‐dose region in the rectum overlapped to the PTV having higher mean and maximum dose.^(27)^ Since the rectal NTCP is sensitive to the high‐dose region where the PTV and rectum overlapped, and the double‐arc technique has a higher mean dose than the single‐arc, the rectal NTCP for the double‐arc technique is therefore higher than the single‐arc. Nevertheless, such increased NTCP is still within the acceptable range when compared to prostate IMRT.^(28)^ In addition, it can be seen in Fig. 3 that there is no dependence of the prostate TCP and rectal NTCP on the prostate volume using the two techniques. For lower rectal dose‐volume criteria ($D_{30\%},D_{50\%},V_{17Gy}$, and $V_{35Gy}$) achieved in the treatment plan, double‐arc technique is still worthwhile to be considered, in spite of the higher rectal NTCP compared to the single‐arc.

## V. CONCLUSIONS

Prostate VMAT plans have been analyzed for five patients using the single‐arc and double‐arc technique. It is found in VMAT plans that the double‐arc technique can lower the dose‐volume criteria of the rectum and bladder (e.g., $D_{30\%},D_{50\%},V_{17Gy}$, and $V_{35Gy}$) but increase the rectal NTCP. The increased rectal NTCP in the double‐arc technique is due to the increase of dose at the high‐dose overlapping region (PTV and rectum), which is sensitive in the NTCP calculation. As the degree of increase of the rectal NTCP is tolerable, it is concluded that the double‐arc technique can effectively decrease the rectal and bladder dose‐volume criteria in a prostate VMAT plan, and is especially crucial when the criteria are critical or difficult to achieve in planning. The increase in the femoral head dose as a cost of improvements in the rectal and bladder dose‐volume criteria is found acceptable in this study.
